# Transcriptome Profiling Analysis of Phosphate-Solubilizing Mechanism of *Pseudomonas* Strain W134

**DOI:** 10.3390/microorganisms10101998

**Published:** 2022-10-10

**Authors:** Shuaibing Wang, Yi Li, Jie Zhang, Xiangying Wang, Jianping Hong, Chen Qiu, Huisheng Meng

**Affiliations:** 1 College of Resources and Environment, Shanxi Agricultural University, Taigu County, Jinzhong 030810, China; 2 College of Life Sciences, Shanxi Agricultural University, Taigu County, Jinzhong 030810, China; 3 College of Urban and Rural Construction, Shanxi Agricultural University, Taigu County, Jinzhong 030810, China

**Keywords:** phosphate-solubilizing bacteria (PSB), transcriptome, organic acids, phosphate-solubilizing mechanisms

## Abstract

Phosphate-solubilizing bacteria (PSB) can alleviate available phosphorus deficiency without causing environmental pollution, unlike chemical phosphate fertilizers. However, the phosphate solubilization mechanisms of PSB are still unclear. Transcriptome sequencing was used to analyze the expression patterns of differential expressed genes (DEGs) of the phosphate-solubilizing bacterium W134 under the conditions of soluble phosphorus (group A), insoluble phosphorus (group B), and lacking phosphorus (group C). Nine DEGs in three different groups were detected by quantitative real-time polymerase chain reaction (qRT-PCR). Then, high performance liquid chromatography (HPLC) was applied to detect the concentrations and composition of organic acids. Compared with group A, Gene Ontology (GO) annotation showed that the cluster of W134 DEGs in groups B and C were basically the same. Besides, the results of enrichment Kyoto Encyclopedia of Genes and Genomes (KEGG) pathway indicated that genes in the Citrate cycle (TCA cycle) pathway closely related to organic acid production were significantly upregulated. The qRT-PCR results were almost consistent with the expression trends of the transcriptome data. The HPLC results showed that the formic acid, ascorbic acid, acetic acid, citric acid, and succinic acid concentrations were significantly increased in group B and C (*p* < 0.05), while the contents of lactic acid and malic acid were significantly increased in group B (*p* < 0.05). The above results provided further validation that the upregulated genes should be related to W134 secretion of organic acids. Our study revealed several potential candidate genes and tried to explain phosphate solubilization mechanisms. This provides a new insight for calcareous reclaimed soil, and it will reduce the need of chemical phosphate fertilizers to promote environmentally friendly agriculture.

## 1. Introduction

Phosphorus (P) is one of the most essential nutrients for plant growth and development, as well as an important component of protoplasts. Many important organic compounds in plants are made of P, which is involved in photosynthesis and important biochemical processes in plants [1,2]. However, P deficiency is a widespread phenomenon in agricultural soils worldwide [3], especially in calcareous soils, where most P forms insoluble calcium phosphate salts with free calcium carbonate due to its unique physicochemical properties [4], the low content of available P that can be absorbed and utilized by crops leads to a huge demand for chemical phosphate fertilizers [4,5]. Global phosphate fertilizers demand reached 45.86 million tons in 2020 according to the reports of the Food and Agriculture Organization of the United Nations [6]. However, the utilization rate of chemical phosphate fertilizer is as low as 10–25% [7,8], and most of it accumulates in the soil. In addition, excessive chemical phosphate fertilizers can lead to soil nutrients imbalance, heavy metal accumulation, and algal bloom [9,10]. Therefore, it is of great significance for agricultural production and environmental protection to excavate the potential P pool and transform insoluble P into available P that can be absorbed and utilized by crops.

Microbes play an indispensable role in soil P cycling, as they mediate bioavailable P in soil [11,12,13]. Due to its high reactivity, P in soils can exist in many inorganic and organic forms that can restrain plant uptake [14]. It has been found that Phosphate-solubilizing bacteria (PSB) can depend on making use of their own metabolites or synergizing with other organisms to transform insoluble P from soil to available P for plant absorption and utilization [15,16,17]. In practice, PSB can also improve P uptake by crops, promote crops growth and enhance soil P cycle through various mechanisms [18,19,20]. Application of PSB in soil can replace or partly reduce the use of inorganic P fertilizer, which may be one of the most efficient and sustainable methods with the lowest cost to protect P resources and prevent P pollution [21,22]. However, the phosphate-solubilization process of PSB is very complex, and the phosphate-solubilization mechanism of PSB is also species-specific. Taking possible effects of the different organic matter, pH, and other characteristics of the soil on the colonization and function of PSB into consideration, it is very necessary to develop suitable PSB for different types of soil [23,24,25].

Calcareous soils, which have stress conditions for PSB growth, are one of the major soil types for crop production in the world [26], and few studies have investigated the isolation of PSB from calcareous soils with high calcium carbonate content [27]. Studies have shown that *Pseudomonas*, one of the PSB, has high phosphate solubility [28,29]. In calcareous soil, the combination of pomace and *Pseudomonas* can effectively increase soil phosphatase activity and soil available P level in a short period of time [30]. In addition, the combined use of wheat straw biochar and *Pseudomonas* can improve the P availability in calcareous soil [31]. Although the above reports have confirmed that *Pseudomonas* can be effectively used in calcareous soil and can improve the availability of P in soil, the mechanism still remains unclear, which largely blocks the application of PSB [32]. Normally, direct oxidation of glucose to gluconic acid is thought to be the main phosphate-solubilizing way in Gram-negative bacteria [33,34]. It has also been found that the phosphate-solubilizing mechanism of PSB is associated with the release of low-molecular-weight organic acids, which are transformed into soluble forms by their hydroxyl and carboxyl chelations to the cation of phosphate [35,36]. Besides, it has been reported that the some DEGs among different P mediums involved in carbon metabolism may play an important role in phosphate solubilizing [37]. At present, there is controversy regarding the effect of P availability on the phosphate-solubilizing ability of PSB [38], and there are few systematic studies on how *Pseudomonas* respond to P-deficiency and synthesize and secrete organic acids. Therefore, in this study, transcriptomics and HPLC were used to analyze the expression patterns of genes from W134 as well as the concentrations and composition of organic acids secreted under three different P-culture conditions. The objectives are as follows: (1) to explore the mechanism of phosphate-solubilization at the transcriptomic level, (2) to determine the kinds of organic acids secreted by W134 under different P-culture conditions, and (3) to verify whether the expression patterns of DEGs under different P-culture conditions is consistent with the transcriptome results of W134 by qRT-PCR.

## 2. Materials and Methods

### 2.1. Strain and Culture Conditions

PSB W134 was isolated from calcareous reclaimed soil by the laboratory of mining soil reclamation and microbial diversity, College of Resources and Environment, Shanxi Agricultural University (GenBank accession numbers: OP485443). It has high phosphorus solubilization capacity (Tables S2 and S3) and is identified as *Pseudomonas sp.* by 16S rRNA-encoding gene sequencing (See the Supplementary Materials for details). To further explore the phosphate-solubilizing mechanism of W134, W134 was inoculated in three different phosphate sources media (Table 1) and grown 28 °C, 160 r/min for two days; three replicates were set for each culture condition. After culture to logarithmic growth period (OD_600_ = 1, effective viable count 2 × 10^8^ CFU mL^−1^), 10 mL of bacteria solution was collected and centrifuged in a high-speed freezing centrifuge (Nison Instrument Co., Ltd., Shanghai, China) for 10 min (12,000 r/min, 4 °C), and the supernatant was filtered through a 0.45-μm membrane and then put into HPLC for the determination of organic acid content. The bacterial cells were washed three times with ultrapure water and stored in the refrigerator at −80 °C for RNA extraction of the transcriptome and qRT-PCR analysis.

### 2.2. RNA Extraction and Library Construction

Total RNA was extracted via a TRIzol-based method (Life Technologies, Carlsbad, CA, USA). Fifty microliters of cell pellet were ground to a fine dry powder in a mortar and pestle under liquid nitrogen. The powder was transferred into an RNase-free tube and vortexed at maximum speed for 15 seconds after 1.3 mL of Trizol was added. The suspension was placed at room temperature for 5 min, and the subsequent steps followed the Trizol extraction method as described by Kang et al. [39]. The NanoPhotometer^®^ spectrophotometer (IMPLEN, Calabasas, CA, USA) was applied to check the RNA purity (OD260/OD280, OD260/OD230). cDNA was synthesized by 1 μg of total RNA as a template. The reverse transcription reaction conditions were set as 25 °C for 10 min, 42 °C for 15 min, and 70 °C for 15 min. Then the sequencing library was constructed using NEBNext^®^ Poly(A) mRNA Magnetic Isolation Module (New England Biolabs, Ipswich, MA, USA) for PCR reaction on the Illumina Novaseq 6000 platform, with pair-end 150 base reads sequencing performed by Genedenovo Biotechnology Co., Ltd. (Guangzhou, China), BioProject number: PRJNA888420.

### 2.3. Clean Reads Filtering and Differentially Expressed Genes Analysis

Raw data were filtered by the flowing standards, (1) removing reads with  ≥10% unidentified nucleotides (N); (2) removing reads with  >50% bases having phred quality scores of ≤20; (3) removing reads aligned to the barcode adapter using FASTP (https://github.com/OpenGene/fastp, version 0.18.0) (accessed on 20 March 2022). Quality trimmed reads were mapped to the reference genome using Bowtie2 [40] (version 2.2.8) allowing no mismatches, and reads mapped to ribosome RNA were removed. Retained reads were aligned with the reference genome (*Pseudomonas lini* BS3782) using Bowtie2 to identify known genes and calculated gene expression by RSEM [41].

The gene expression level was further normalized by using the fragments per kilobase of transcript per million (FPKM) mapped reads method to eliminate the influence of different gene lengths and amount of sequencing data on the calculation of gene expression. The edgeR package (http://www.r-project.org/) (accessed on 12 April 2022) was used to identify differentially expressed genes (DEGs) across samples with fold changes ≥2 and a false discovery rate-adjusted P (*q* value) < 0.05. DEGs were then subjected to an enrichment analysis of GO function and KEGG pathways, and *q* values were corrected using <0.05 as a threshold.

### 2.4. qRT-PCR Analysis

The methods of RNA extraction and reverse transcription have been described above (2.2). The 10 μL RT reaction mix was then diluted × 10 in nuclease-free water. Real-time PCR was performed using LightCycler^®^ 480 II; Real-time PCR Instrument (Roche, Swiss). PCR procedure: 94 °C 30 s, 94 °C 5 s, 60 °C 30 s, 45 cycles. At the end of the PCR cycles, melting curve analysis was performed to validate the specific generation of the expected PCR product. Using the 2^−∆∆Ct^ method [42], three biological replicates per sample, three technical replicates per biological replicate, and 16S rRNA-encoding gene was used as internal reference. The primer sequences were designed in the laboratory and synthesized by TsingKe Biotech based on the mRNA sequences obtained from the NCBI database as follows (Table 2):

### 2.5. Determination of Organic Acids

Peak time of each organic acid was determined on a Shimadzu LC-20AD high-performance liquid chromatography (HPLC, Shimadzu Technologies, Kyoto, Japan); the following organic acids were measured: acetic acid; ascorbic acid; citric acid monohydrate; formic acid; L-(+)-lactic acid; L-(−)-malic acid; propionic acid; succinic acid; and DL-tartaric acid. The concentration gradients of 10, 20, 50, 100, and 200 μg mL^−1^, depending on the organic acid concentration and peak area, make a standard curve for each organic acid. The supernatant was obtained as described above (2.2). The chromatographic separation conditions were as follows: Acclaim™ 120 C18 column (4.6 mm × 250 mm), mobile phase 98% phosphoric acid and 2% acetonitrile solution, pH 2.70, UV detection wavelength 210 nm, flow rate 1 mL min^−1^, injection volume 20 μL, column temperature 35 °C. According to the HPLC results, the kinds of organic acids were identified, and the contents of organic acids were calculated.

### 2.6. Statistical Analysis

Microsoft Excel 2010 (Microsoft, Redmond, WA, USA) was used to collate the data. RStudio (version 3.6.2) “agricolae” package was used for One-way analysis of variance (ANOVA), and Duncan’s test were used to compare the means for each environmental variable, with a significance level of *p* < 0.05. Series test of cluster was performed with Short Time-Series Expression Miner (version 1.3.13), and gene expression was preprocessed using log2 normalization to generate the most representative modules. The *p* value is used to measure the relationship between the number of genes in the module and the expected value of the random distribution.

## 3. Results

### 3.1. Transcriptome Sequence Analysis of PSB W134

After data filtering, 15,079,219; 15,900,898; and 15,481,295 clean reads were obtained, respectively, with Q30 of 95.86, 95.71, and 95.51 (Table S4). We plotted the correlation coefficients within and between groups into a heatmap (Figure S1), in which the high correlation of gene expression among repeated samples in groups A, B, and C could be found. Generally, the depth and quality of the sequencing data in this experiment were qualified, and the assay has good repeatability among intra-groups, which allows further quantitative analysis of gene expression obtained from sequencing.

### 3.2. Statistics of DEGs

**Figure 1:**
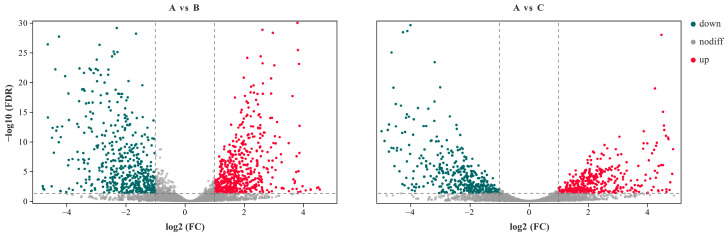
Differential genes expression of W134. Differential genes were screened using statistical tests for FDR values and log2 FC, with a threshold of FDR < 0.05, |log2 FC| > 1. log2 FC: the value of the fold change (FC) of the current comparison group after log2. A: soluble P; B: insoluble P; C: lacking P. down: down-regulated; up: up-regulated; nodiff: no differential.

**Figure 2:**
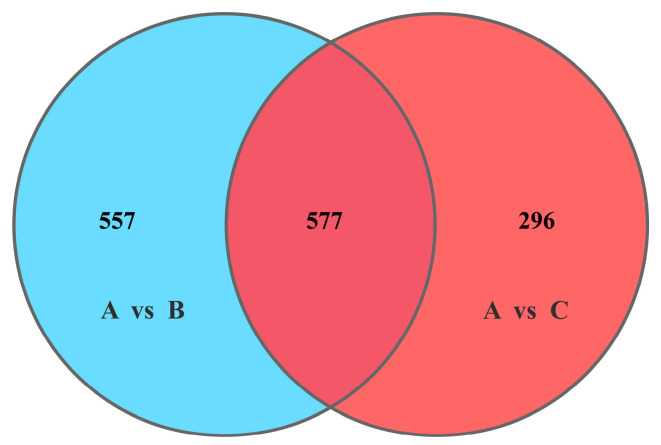
Overlap of DEGs. A: soluble P; B: insoluble P; C: lacking P.

There were 5580 genes in the reference genome of *Pseudomonas lini* BS3782. The RNA reads ribosomal removal were aligned to the reference genome through the mapping software Bowtie2. The mapping ratios were 85.35% for group A, 85.17% for group B, and 89.81% for group C. Subsequently, the difference of gene expression under different P source conditions was analyzed by DESeq2 software according to the screening condition of false discovery rate (FDR) ≤ 0.05. The results showed that 585 genes were upregulated and 549 genes were downregulated in A vs. B, while 455 genes were upregulated and 418 genes were downregulated in A vs. C (Figure 1). Furthermore, there were 577 consensus DEGs in the two differentially expressed groups, 557 unique genes in the A vs. B comparison group, and only 296 unique genes in the A vs. C comparison group (Figure 2).

**Figure 3:**
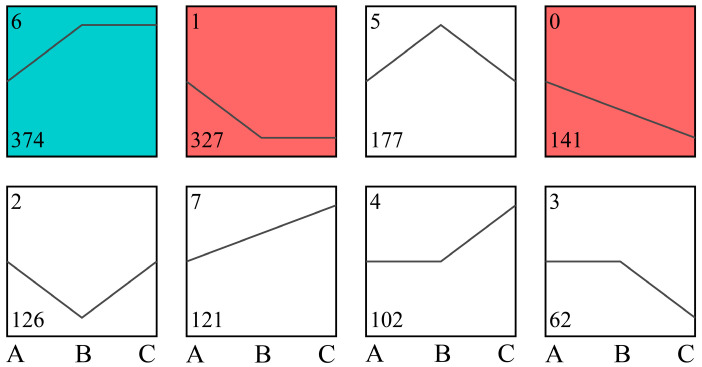
Trends of the differential genes in B and C by series test of cluster analysis. The trend profiles with color indicated significant enrichment (*p* < 0.05), and different color indicated different enrichment trends. No color trend profiles indicated no significant enrichment trends. A: soluble P; B: insoluble P; C: lacking P.

According to the above results (Figure 1), the differential genes in groups B and C were sorted in the order of soluble-insoluble-lacking-P (A-B-C), and series tests of cluster were performed. Gene expression patterns were divided into eight profiles (Figure 3), sorted by the number of enriched genes from large to small, with the ID of the trend at the top left of each profile and the number of genes in the profiles at the bottom. The results showed that the genes in profile 6 were significantly more expressed in groups B and C than in group A (*p* < 0.05), indicating that the expression of these genes was higher in insoluble-P as well as lacking-P conditions than in soluble-P conditions; the genes expression of profiles 1 and 0 in group A was higher than in group B and C (*p* < 0.05), which indicated that the gene expression of profile 1 and 0 was higher in soluble-P than in insoluble-P and lacking-P.

### 3.3. GO Analysis of DEGs

**Figure 4:**
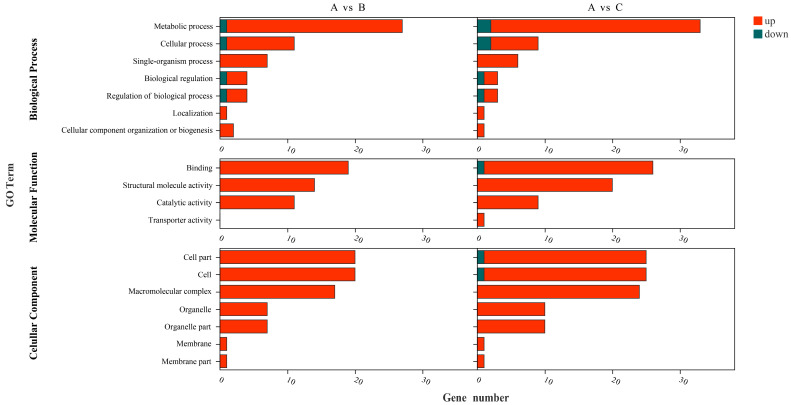
Gene ontology (GO)-terms analysis. A: soluble P; B: insoluble P; C: lacking P.

GO enrichment analysis was performed for the DEGs (Figure 4), with *q*-value ≤ 0.05 as the criterion, and W134 DEGs were assigned to the three main GO terms, namely “Biological Process”, “Molecular Function”, and “Cellular Component”.

Based on the annotation of DEGs in groups A and B, most genes were related to metabolic process in Biological Process, binding in Molecular Function, and cell part. The cell in Cellular Component was upregulated in group B, and metabolic process was the major category of upregulated genes. The differences between groups A and C were abundant. Most of the genes upregulated in group C in Biological Process were related to metabolic Process, while the genes upregulated in group C were related to binding and structural molecule activity in Molecular Function, and the Cellular Component is related to the cell part, cell, macromolecular complex. Metabolic process, binding, cell part, and cell are the major classes of genes upregulated in group C.

These differences directly or indirectly reflect the response of W134 to low P stress. Among the DEGs, we focused on those that showed uniform and continuous changes with the decrease of exogenous phosphate concentration.

### 3.4. KEGG Analysis of DEGs

**Figure 5:**
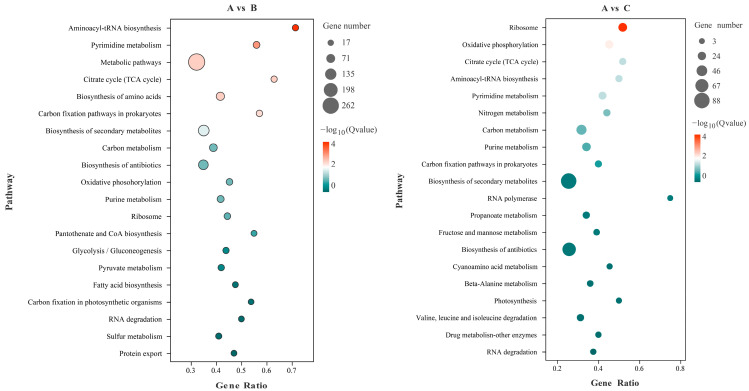
KEGG analysis of DEGs in W134 B and C groups. The significantly enriched top 20 pathway was selected based on FDR < 0.05. A: soluble P; B: insoluble P; C: lacking P.

KEGG enrichment analysis was carried out for the different genes from different P source conditions, and the top 20 metabolic pathways with the most significant enrichment were listed (Figure 5). Among them, the metabolic differences in transcriptome expression between groups A and B were mainly concentrated in aminoacyl-tRNA biosynthesis, pyrimidine metabolism, metabolic pathways, carbon fixation pathways in prokaryotes, citrate cycle (TCA cycle), etc. The differences of transcriptome expression between groups A and C mainly focus on ribosome, oxidative phosphorylation, citrate cycle (TCA cycle), and nitrogen metabolism. By further studying the metabolic pathways involved in the DEGs, we found that the DEGS in groups B and C were significantly enriched in the Citrate cycle (TCA cycle) pathway, which is closely related to organic acids production, the results suggest that organic acids play an important role in the process of insoluble-P and lacking-P, and the genes involved in the Citrate cycle (TCA cycle) pathway may enhance W134’s ability to secrete organic acids under low P stress.

### 3.5. Genes Expression in the Citrate Cycle Pathway

**Figure 6:**
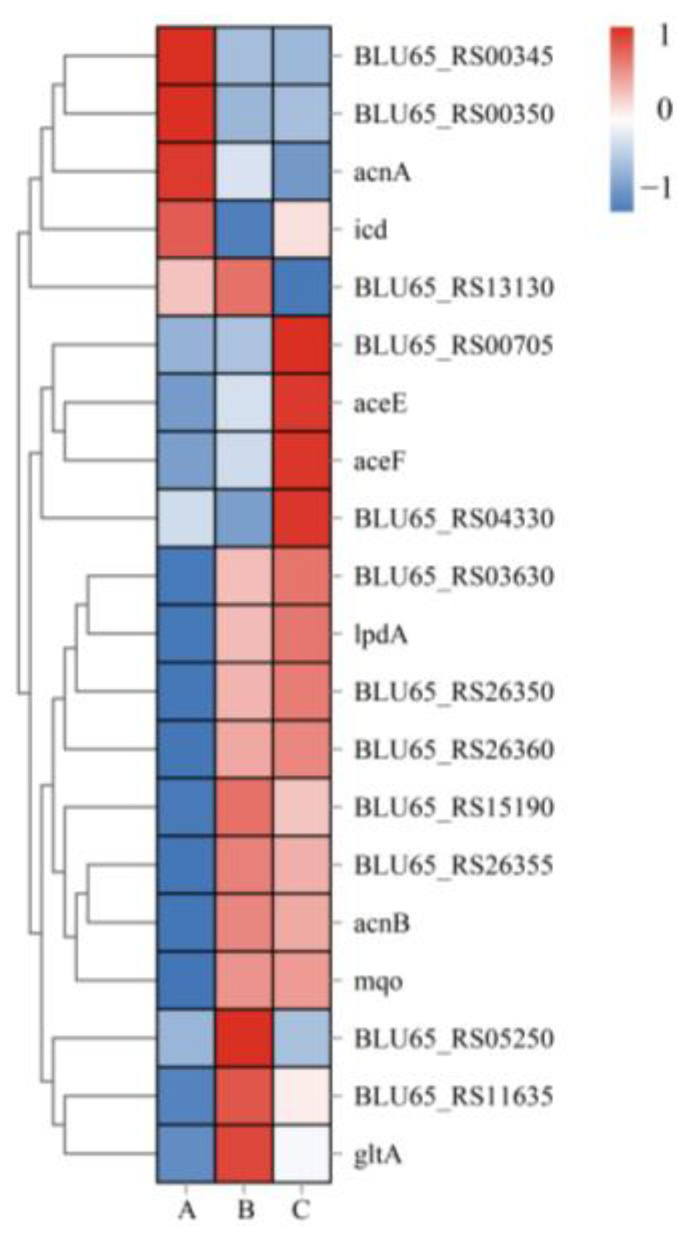
Heat map of target genes in the citrate cycle. A: soluble P; B: insoluble P; C: lacking P.

Based on the results of DEGs analysis, the target genes were selected, and the heatmap was drawn according to the expression level in the samples. Figure 6 summarizes the expression of genes in the citrate cycle (TCA cycle) pathway under different P sources, excluding seven genes with extremely low expression under the three P sources, resulting in twenty genes. The results showed that the expression levels of BLU65_RS05250, BLU65_RS11635, and gltA in group B were higher than in groups A and C, while the expression levels of BLU65_RS00705, aceE, aceF, and BLU65_RS04330 in group C were significantly higher than in the other two conditions. In addition, it was found that the expression trends of BLU65_RS03630 and the other eight genes were similar under groups B and C.

In the Citrate cycle (TCA cycle) pathway (Table 3), 18 genes were significantly upregulated (at least to a significant level in one group), compared with 17 genes up-regulated and 1 gene down-regulated in the other two groups. Further analysis of significantly upregulated genes revealed that BLU65_RS00350, BLU65_RS05250, gltA, and oadA were significantly upregulated only in group B. Among them, BLU65_RS05250 and oadA were both related to pyruvate carboxylase (the only difference was their subunits), and pyruvate carboxylase may play an important role in the solubilization and uptake of insoluble-P. The gene significantly upregulated only in group C was aceF, a class of dihydrolipoyllysine-residue acetyltransferase, suggesting that the conversion of pyruvate to Acetyl-CoA and CO_2_, which generates energy to participate in the TCA cycle, may accompany the PSB W134 under lacking-P conditions.

### 3.6. qRT-PCR Validation

**Figure 7:**
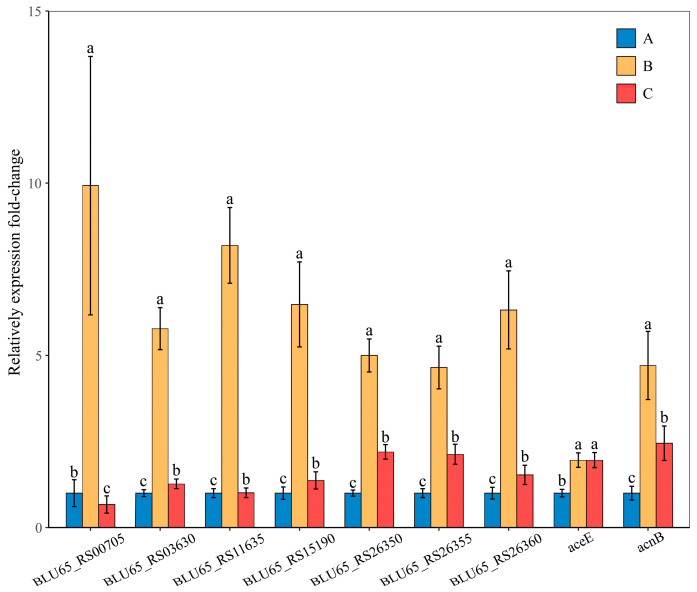
Analysis of gene expression difference in Citrate cycle (TCA cycle) pathway by qRT-PCR. The different letters indicate that the data of the same row are significantly different at the 0.05 level. A: soluble P; B: insoluble P; C: lacking P.

To verify the reliability of the transcriptome data, the data were validated through qRT-PCR analysis, and nine genes were selected from the Citrate cycle (TCA cycle) pathway; Class II fumarate hydratase (BLU65_RS00705) was upregulated in both B and A groups, and the remaining selected genes were significantly higher in both the B and C groups than in group A (Figure 7). Data from qRT-PCR were consistent with those obtained from RNA-seq.

### 3.7. Determination of Organic Acids Contents

**Figure 8:**
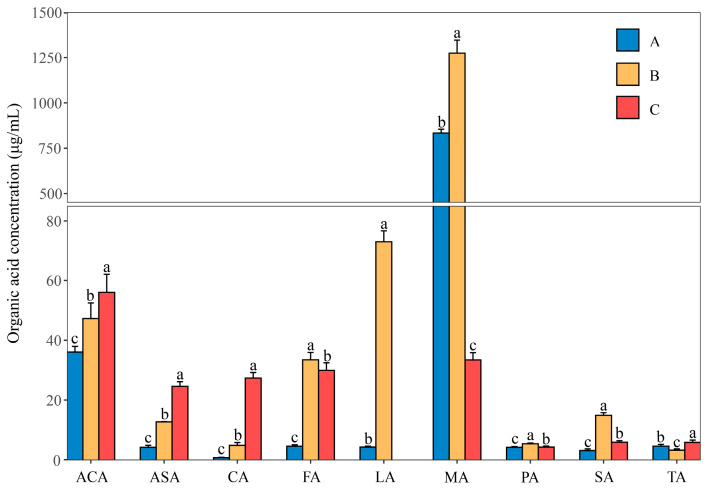
Concentration of organic acids secreted by W134 under different phosphorus source conditions. ACA: Acetic acid; ASA: Ascorbic acid; CA: Citric acid monohydrate; FA: Formic acid; LA: L-(+)-Lactic acid; MA: L-(−)-Malic acid; PA: Propionic acid; SA: Succinic acid; TA: DL-Tartaric acid. The different letters indicate that the data of the same row are significantly different at the 0.05 level. A: soluble P; B: insoluble P; C: lacking P.

RNA-seq and qRT-PCR analysis showed that the key genes of W134 and organic acids production were significantly up-regulated in groups B and C, but the exact organic acid was not studied in detail; the composition and content of organic acids were analyzed by HPLC (Figure 8).The results showed that compared with group A, the contents of formic acid, ascorbic acid, acetic acid, citric acid, and succinic acid were significantly increased in groups B and C (*p* < 0.05), and propionic acid was significantly increased in group B (*p* < 0.05); the content of lactic acid in group B was significantly higher than in group A (*p* < 0.05), the content of tartaric acid in group C was significantly higher than in group A and group B (*p* < 0.05), and the content of malic acid increased significantly in group B (*p* < 0.05).

## 4. Discussion

Transcriptome sequencing revealed that the GO terms of the DEGs of PSB W134 was essentially the same under the culture conditions of insoluble-P (group B) and lacking-P (group C) compared with soluble-P (group A) (Figure 4); these results suggest that W134 may have undergone low P stress during the initial stage of culture in both groups B and C, and the genes changed simultaneously in groups B and C may be involved in low P stress induction. However, the gene functions of the significantly enriched genes in groups B and C were slightly different in the series test of cluster analysis (Figure 3), suggesting that the genes that changed in both groups may be involved in the induction of low P stress, the difference between the two genes should be a potential key gene involved in the degradation of insoluble P. Based on this hypothesis, insoluble phosphate degradation genes may be associated with the metabolic process in biological process, mainly localized to cell part and cell, involved in transport, and structural molecule activity and other biological functions (Figure 4); more precise results are yet to be confirmed by follow-up studies. The TCA cycle produces a variety of organic acids, such as citric acid, α-ketoglutaric acid, succinic acid, and malic acid [43,44]. The acidolysis pathway is considered to be a major aspect of the phosphate-solubilizing mechanism, many organic acids, including citric acid, formic acid, D-gluconic acid acid and oxalic acid, participate in microbial solubilization of insoluble inorganic P [33,34,36]. the results of KEGG enrichment in this study suggest that genes in the Citrate cycle (TCA cycle) pathway, which is closely related to organic acids production, are up-regulated (Figure 5 and Figure 6, Table 3). The HPLC results showed that the concentrations of all organic acids in group B were higher than those in group A except for tartaric acid (Figure 8), which corresponded to the transcriptome results. This may be due to the fact that W134, under low P stress, dissolves insoluble P by secreting large amounts of organic acids, similar to the present study [37]. In addition, a large amount of malic acid (1274.38 μg mL^−1^) was found in group B, which was significantly higher than those in the other two groups and the other acids. It dissolves insoluble P in the environment mainly by secreting malic acid. Whereas, a study in the PSB *Burkholderia multivorans* WS-FJ9 found that pyruvate was the main organic acid secreted when solubilizing P [38]. Another study in the PSB *Enterobacter cloacae* RW8 found that lactic acid, succinic acid, and citric acid were the main organic acids secreted [45]. These results showed that different PSB would secrete the varied types and concentrations of organic acids to solubilize P. The mechanism of P degradation by PSB may be changed by the change of environment and metabolic pathway [46].

Recent advances in the field of biofertilizers have discovered novel elite strains such as *Pseudomonas plecoglossicida* isolated from soybean rhizosphere, which solubilized 75.39 mg L^−1^ phosphorus and also produced plant growth hormones such as indole acetic acid up to 38.89 ppm [47]. The solubility of tricalcium phosphate in W134 was 587.52 mg L^−1^ (Table S2), which was 679.31% higher than that of *Pseudomonas plecoglossicida*. Studies have shown that the combination of PSB *Pseudomonas mallei* and *Pseudomonas cepaceae* with nano-P (0.1 g L^−1^) can improve crop yield, chlorophyll content, and antioxidant enzyme activity in calcareous soil [48]. Application of Fe-EDTA with PSB *Pseudomonas putida* P159, *Pseudomonas fluorescens* T17-24, and *Bacillus subtilis* P96 improved root shoot biomass and nutrition of *Sorghum bicolor* in low fertility calcareous soil [49]. W134 was screened from calcareous reclaimed soil, and the pH of three different phosphate media used in this study was also slightly alkaline. The results showed that *Pseudomonas* could play an effective role in phosphate-solubilization in an alkaline environment, and the results of this study showed that the levels of formic acid, L-(+)-lactic acid, L-(−)-malic acid, propionic acid, and succinic acid in group B were significantly higher than those in group A and C, indicating that PSB W134 could reduce pH by secreting organic acid under the condition of partial alkaline culture. This is beneficial to the dissolution of insoluble phosphate in calcareous soil [6,50,51]. In addition, L-(+)-lactic acid was not detected in group C, while L-(+)-lactic acid was the second most abundant of the nine organic acids in group B, and a small amount of L-(+)-lactic acid was found in group A. The results indicated that different P sources had influence on the types of organic acids secreted by W134.

Transcriptome analysis revealed that BLU65_RS00350, BLU65_RS05250, gltA, and oadA were significantly upregulated only in group B (Table 3), and BLU65_RS05250 and oadA were associated with pyruvate carboxylase, which can be converted to organic acids by the pyruvate metabolic pathway, such as acetic acid and lactic acid [52]. This may be the reason why L-(+)-lactic acid was not detected in group C. Both BLU65_RS26355 and BLU65_RS26360 were succinate dehydrogenase, which was significantly increased in groups B and C, resulting in a significantly higher succinic acid content than in group A. Succinate dehydrogenase is the only enzyme that is involved both in the TCA cycle and in respiration via the electron transport chain [53], so we carried on the qRT-PCR. In addition, the total variation trend of differential genes in the citrate cycle (TCA cycle) pathway was the same in group B and group C, but a few genes were only differentially expressed in group B or group C. The results indicated that it may be involved in the induction of low P stress and the degradation of insoluble P. qRT-PCR results showed that the expression patterns of the upregulated differential genes in group B and group C were similar to those in the transcriptome. In summary, PSB W134 upregulates gene expression in the organic acids synthesis pathway and secretes more organic acids, such as malic acid, lactic acid, and acetic acid, in the insoluble-P environment. The expression patterns and secretion of organic acids of the relevant genes in PSB W134 are different from some PSB reported previously, suggesting that different PSB have different p-solubilization mechanisms. This study has certain reference significance for other bacteria to carry out similar research and clarify the P-releasing mechanism of PSB W134 under different P source conditions. We should pay attention to the actual application effect of PSB in the follow-up study and carry out pot and field experiments to further verify the mechanism of phosphate-solubilization, so as to provide highly effective and adaptable strain of PSB for calcareous soil.

## 5. Conclusions

Transcriptome analysis showed that the Citrate cycle (TCA cycle) pathway genes of PSB W134 was upregulated and produced more organic acids, mainly malic acid (1274.38 μg mL^−1^). This results in the dissolution of insoluble P. qRT-PCR and showed that the key genes of organic acid synthesis were upregulated in the insoluble phosphate group, which further confirmed that the upregulated genes induced W134 to secrete organic acids. Among them, BLU65_RS00705, BLU65_RS11635, aceE, and other genes may be the key genes involved in regulating the secretion of organic acids in PSB W134.

## Figures and Tables

**Table 1:** Different media with different phosphorus sources.

Three Different Media	Medium Formulation	pH
Soluble P (Group A)	Glucose 10 g, KH_2_PO_4_ 4.3874 g, (NH_4_)_2_SO_4_ 0.5 g, NaCl 0.3 g, KCl 0.3 g, MgSO_4_ • 7H_2_O 0.03 g, FeSO_4_ • 7H_2_O 0.03 g, MnSO_4_ • H_2_O 0.03 g, aseptic water 1000 mL	7.80
Insoluble P (Group B)	Glucose 10 g, Ca_3_(PO_4_)_2_ 5 g, (NH_4_)_2_SO_4_ 0.5 g, NaCl 0.3 g, KCl 0.3 g, MgSO_4_ • 7H_2_O 0.03 g, FeSO_4_ • 7H_2_O 0.03 g, MnSO_4_ • H_2_O 0.03 g, aseptic water 1000 mL	7.80
Lacking P (Group C)	Glucose 10 g, CaCl_2_ 5.3678 g, (NH_4_)_2_SO_4_ 0.5 g, NaCl 0.3 g, KCl 0.3 g, MgSO_4_ • 7H_2_O 0.03 g, FeSO_4_ • 7H_2_O 0.03 g, MnSO_4_ • H_2_O 0.03 g, aseptic water 1000 mL	7.80

**Table 2:** Primers used in qRT-PCR analysis.

Unigene	Forward Primer	Reverse Primer
BLU65_RS00705	5′-ACAGATCAGCGAATCCCAA-3′	5′-GGAAGTGCTGCATGAAGTC-3′
BLU65_RS03630	5′-CCGTTACCTGATCGGTGAC-3′	5′-GTCGATGCACTTGGCATAG-3′
BLU65_RS11635	5′-GTGATCAAGCAAGACGACC-3′	5′-CCTGGATGAAATCCACGG-3′
BLU65_RS15190	5′-CCCGCTCGAAGATCATCTATAC-3′	5′-AAAGCCTCTACGATAGGCA-3′
BLU65_RS26350	5′-CTCTACCTGCACGACCCTAA-3′	5′-CGAAACATCGGTGACAGT-3′
BLU65_RS26355	5′-GTTGATACCGGTGGTAAAGAC-3′	5′-GAGCGACGATAGGAGAAAC-3′
BLU65_RS26360	5′-TGCCGATCCGAACGATGA-3′	5′-ATGTACTCGATAGCGTCCT-3′
aceE	5′-CAAAGAAGGCGAAGACCG-3′	5′-TGGTGATGGCGTAAGGGA-3′
acnB	5′-GGTGGCTACAACATCGTG-3′	5′-GTGGAAGGCATCGAACATCA-3′
16S rRNA	5′-GGGGAGTACGGTCGCAAGAT-3′	5′-CATGTCAAGGGTAGGTAAGGTTT-3′

16S rRNA has the advantages of small species, high content (about 80% of bacterial DNA content), moderate molecular size, and existence in all organisms. It can not only reflect the differences between different bacterial genera, but also be easily obtained by sequencing technology, which has high conservation in structure and function.

**Table 3:** Different genes of Citrate cycle (TCA cycle) pathway.

Gene ID	Log2 FC		Significant		FDR		Description
	B/A	C/A	B/A	C/A	B/A	C/A	
BLU65_RS00350	−1.64	−1.53	yes	no	1.41 × 10^−2^	1.16 × 10^−1^	alpha-ketoacid dehydrogenase subunit beta
BLU65_RS00705	1.87	5.13	yes	yes	1.83 × 10^−4^	2.28 × 10^−5^	class II fumarate hydratase
BLU65_RS03630	1.56	1.80	yes	yes	4.94 × 10^−6^	4.69 × 10^−3^	phosphoenolpyruvate carboxykinase (ATP)
BLU65_RS05250	1.53	0.15	yes	no	3.40 × 10^−7^	9.61 × 10^−1^	pyruvate carboxylase subunit A
BLU65_RS11635	2.24	1.66	yes	yes	4.63 × 10^−9^	9.35 × 10^−3^	fumarate hydratase
BLU65_RS15190	2.41	2.09	yes	yes	2.70 × 10^−14^	6.88 × 10^−4^	isocitrate dehydrogenase
BLU65_RS26350	2.11	2.32	yes	yes	1.28 × 10^−9^	4.87 × 10^−5^	2-oxoglutarate dehydrogenase
BLU65_RS26355	1.64	1.49	yes	yes	5.02 × 10^−8^	3.97 × 10^−3^	succinate dehydrogenase
BLU65_RS26360	1.45	1.55	yes	yes	8.56 × 10^−6^	7.68 × 10^−3^	succinate dehydrogenase
aceE	1.18	2.39	yes	yes	2.94 × 10^−3^	1.41 × 10^−4^	pyruvate dehydrogenase (acetyl-transferring), homodimeric type
aceF	0.61	1.56	no	yes	1.01 × 10^−1^	3.16 × 10^−2^	dihydrolipoyllysine-residue acetyltransferase
acnB	2.56	2.42	yes	yes	5.15 × 10^−14^	1.12 × 10^−5^	aconitase
gltA	1.82	1.11	yes	no	2.48 × 10^−6^	8.55 × 10^−2^	citrate synthase I, hexameric type
oadA	2.32	0.86	yes	no	1.76 × 10^−14^	1.66 × 10^−1^	pyruvate carboxylase subunit B
odhB	1.69	1.53	yes	yes	2.38 × 10^−7^	9.35 × 10^−3^	2-oxoglutarate dehydrogenase complex dihydrolipoamide succinyltransferase
sdhC	1.50	1.58	yes	yes	1.89 × 10^−5^	5.42 × 10^−3^	succinate dehydrogenase
sucC	1.78	1.78	yes	yes	4.87 × 10^−6^	2.97 × 10^−3^	Succinyl-CoA ligase [ADP-forming] subunit beta
sucD	2.48	2.12	yes	yes	5.97 × 10^−13^	2.99 × 10^−4^	succinyl-CoA synthetase, alpha subunit

Yes/No stand for a significant/insignificant difference were detected. Log2 FC: the value of the fold change (FC) of the current comparison group after log2. A: soluble P; B: insoluble P; C: lacking P.

## Data Availability

Not applicable.

## Supplementary Materials

The following supporting information can be downloaded at: https://www.mdpi.com/article/10.3390/microorganisms10101998/s1, Figure S1: Heatmap of gene expression level correlation; Figure S2: Phylogenetic tree based on 16S rRNA gene sequences. The relationships among the PSB strains and between representatives of other related taxa were showed; Table S1: The consist of PCR reaction system; Table S2: The available phosphate content of phosphate-solubilizing bacteria in insoluble phosphorus medium; Table S3: The diameter of PSB W134 colony and phosphate-solubilizing halo on Ca_3_(PO_4_)_2_ medium. Table S4: W134 transcriptome sequencing quality assessment. PSB W134 screening and identification: The screening and identification process of W134 strain was described in detail. References [54,55,56] are cited in the Supplementary Materials.
