# Supplementary material for: Transcriptome Profiling Analysis of Phosphate-Solubilizing Mechanism of Pseudomonas Strain W134
Source: Microorganisms. 2022 Oct 10;10(10):1998. doi: 10.3390/microorganisms10101998 (PMC9609647; doi:10.3390/microorganisms10101998)
Supplement: Supplementary file 1 [file microorganisms-10-01998-s001.zip › microorganisms-1895827-supplementary.pdf]

PSB W134 screening and identification:

The calcareous reclaimed soil samples were taken from the Wujinshan mining area, Hougou Village, Jinzhong Province, China (37°50' N, 112°48' E), screened from calcareous reclaimed soil by Laboratory of Mining Soil Reclamation and Microbial Diversity, College of Resources and Environment, Shanxi Agricultural University. Soil samples (2.0 g) and sterile water (30 ml) were mixed to obtain a bacterial suspension. Bacterial strains were cultured and purified on Luria-Bertani medium according to the dilution coating method and streak plate method [1]. The bacteria with P-solubilizing circles were isolated and cultured in the National Botanical Research Institute's Phosphate (NBRIP) growth medium [2]. The phosphate-solubilizing ability of the strain in NBRIP medium was analyzed, and then the strain with the phosphate-solubilizing ability higher than 200 mg L^{-1} was cultured in three different mediums of insoluble P source, the effective phosphorus content of PSB W134 in three different mediums (Molybdenum blue method [3]) and the diameter of P-solubilizing circles were determined (Table S2, Table S3).

Bacterial genomic DNA was extracted according to the manual of SK1201-UNIQ-12 column bacterial genomic DNA extraction kit, and 16S rRNA-encoding gene sequencing was performed. The primer sequence was: primer 1: 7F (5′-CAGAGTTTGATCCTGGCT-3′); primer 2:1540R (5′-AGGAGGTGATCCAGCCGCA-3′), designed in the laboratory and synthesized by Beijing TsingKe Biotechnology Co., Ltd (Beijing, China). The PCR amplification system was 25 μL and the reaction system was as follows (Table S1). The DNA sequences obtained from W134 16S rRNA-encoding gene sequencing were entered into GenBank, and all sequences in GenBank were compared with BLAST program. A phylogenetic tree was constructed using the neighbor-joining algorithm with MEGA (version 11.0.13). The identification of PSB strains based on 16S rRNA sequences and their phylogeny were presented in Fig. S2. The sequence similarity between W134 and *Pseudomonas sp.* was the highest.

Table. S1. The consist of PCR reaction system

Ingredient	Content	Ingredient	Content
Template	1 μL	dNTP	0.5 μL
Primer 1	0.5 μL	Taq (5μ μL^{-1})	0.2 μL
Primer 2	5 μL	ddH2O	19.8 μL
	10*Taq reaction Buffer		2.5 μL

Table. S2. The available phosphate content of phosphate-solubilizing bacteria in insoluble phosphorus medium

Medium		The content of available phosphorus (mg L^{-1})	The ability of dissolving phosphorus (mg L^{-1})
$Ca_3(PO_4)_2$	Without PSB	30.41 ± 0.12	31.01 ± 0.75
	PSB W134	591.51 ± 3.84	587.52 ± 9.44
$FePO_4$	Without PSB	48.31 ± 1.38	—
	PSB W134	169.54 ± 5.89	100.13 ± 7.31
$AlPO_4$	Without PSB	35.69 ± 0.58	—
	PSB W134	92.21 ± 6.33	55.17 ± 2.21

$Ca_3(PO_4)_2$: glucose 10 g, $Ca_3(PO_4)_2$ 5 g, $(NH_4)_2SO_4$ 0.5 g, NaCl 0.3 g, KCl 0.3 g, $MgSO_4 \cdot 7H_2O$ 0.03 g, $FeSO_4 \cdot 7H_2O$ 0.03 g, $MnSO_4 \cdot H_2O$ 0.03 g, sterile water 1000 mL; $FePO_4$: $FePO_4$ was used to replace $Ca_3(PO_4)_2$ by equimolar PO_4^{3-}; $AlPO_4$: $AlPO_4$ was used to replace $Ca_3(PO_4)_2$ by equimolar PO_4^{3-}.

Table. S3. The diameter of PSB W134 colony and phosphate-solubilizing halo on $Ca_3(PO_4)_2$ medium

	d (cm)	D (cm)
W134	0.92 ± 0.10	2.31 ± 0.33

d: colony diameter; D: diameter of phosphate-solubilizing halo

Table. S4. W134 transcriptome sequencing quality assessment

Sample	Clean reads num	Read length	Q20 (%)	Q30 (%)	GC (%)
A1	14396752	150 + 150	98.62%	95.77%	54.61%
A2	15437524	150 + 150	98.59%	95.70%	54.97%
A3	15403380	150 + 150	98.73%	96.10%	55.05%
B1	14968988	150 + 150	98.74%	96.11%	54.93%
B2	14374108	150 + 150	98.80%	96.23%	54.95%
B3	18359598	150 + 150	98.28%	94.79%	54.78%
C1	14111452	150 + 150	98.82%	96.20%	52.60%
C2	19642912	150 + 150	97.98%	94.18%	55.35%
C3	12689520	150 + 150	98.77%	96.16%	55.34%

A: soluble P; B: insoluble P; C: lacking P. down: down-regulated; up: up-regulated.

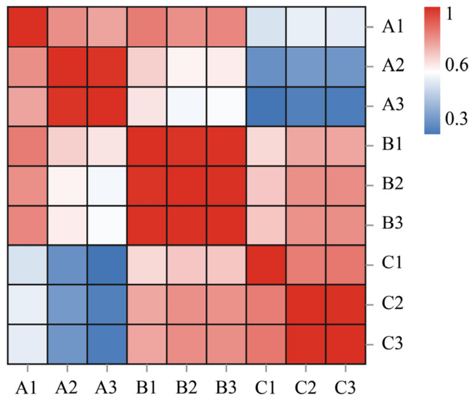

Figure S1. Heatmap of gene expression level correlation

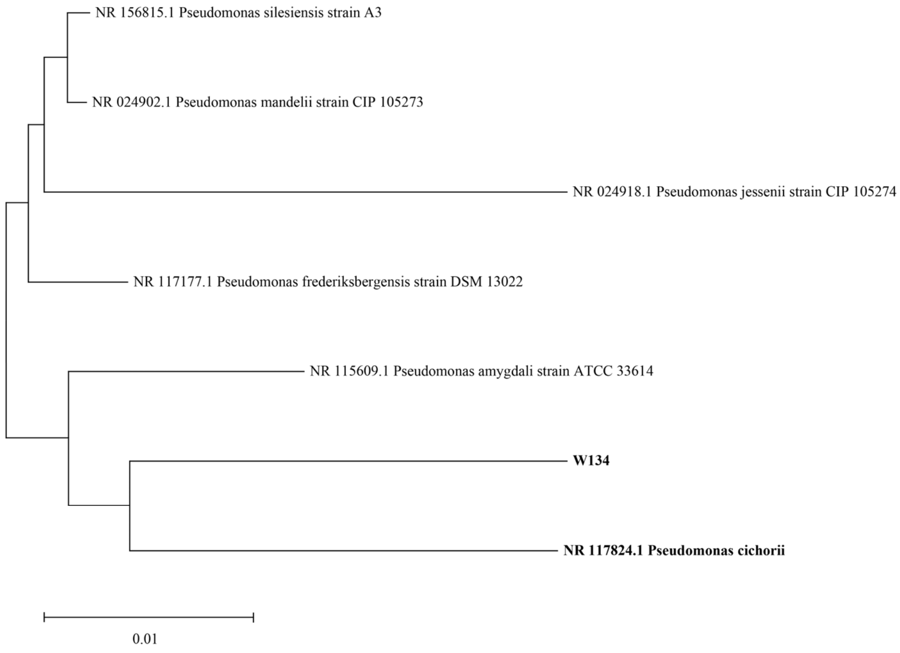

Figure S2. Phylogenetic tree based on 16S rRNA gene sequences. The relationships among the PSB strains and between

representatives of other related taxa were showed.

1. Sanders, E.R. Aseptic laboratory techniques: plating methods. *JoVE (Journal of Visualized Experiments)* **2012**, e3064, doi:https://doi.org/10.3791/3064.
2. Nautiyal, C.S. An efficient microbiological growth medium for screening phosphate solubilizing microorganisms. *FEMS microbiology Letters* **1999**, *170*, 265-270, doi:https://doi.org/10.1111/j.1574-6968.1999.tb13383.x.
3. Murphy, J.; Riley, J.P. A modified single solution method for the determination of phosphate in natural waters. *Analytica chimica acta* **1962**, *27*, 31-36, doi:https://doi.org/10.1016/S0003-2670(00)88444-5.
